# Vaccination: A Consideration of Some Points as to the Identity of Variola and Vaccinia

**Published:** 1881-10

**Authors:** Thomas F. Wood


### ARTICLE III.

**VACCINATION: A CONSIDERATION OF SOME POINTS AS TO THE IDENTITY OF VARIOLA AND VACCINIA. BY THOMAS F. WOOD.**

The physician of the present day is no longer an anxious inquirer about the minute details of the theory and practice of vaccination. He regards all the difficulties as having been overcome, and he is willing to rest upon the glories which Jenner achieved. Notwithstanding the apparently settled state in which we find vaccination to-day, there are few more intricate studies underlying any subject than the present practice of vaccination.

The original works of Jenner, Woodville, Pearson, Bryce, Willan, Steinbrenner, Gregory, Bonsquet, Waterhouse, Redmond Coxe, and the hundred other carefully prepared volumes which from 1798 to 1843 were read and studied with so much anxiety, are fast disappearing from private libraries, and they are only known to the reader of to-day by the meagre extracts made from them, which are found in the current standard text-books. The literature of vaccination embraced within the dates mentioned above, marked the most brilliant era in medicine, and no richer field of study can be found to-day, than the treatises on vaccination prepared by so many careful writers. He who would be master of the vast questions which go to make up our knowledge of vaccination, cannot do so and neglect the original works of Jenner and his cotemporaries. Many of the principles there wrought out stand fast to-day. Many of the questions then anxiously propounded remain undetermined to-day. Many of the fast rules laid down by Jenner, and neglected by a few generations, we gladly return to eighty years after they are announced, because by disregarding them we have been led into error.

We wish we could feel as confident as Professor Lyman that recent studies of the nature of vaccine (CHICAGO MEDICAL JOURNAL AND EXAMINER, July, 1881, p. 1) have given any material weight to the doctrine of identity. He thinks "we are now in a position to form a clear idea regarding a subject in connection with which there is no longer any place for mystery."

He furthermore says, in the same article:

"The opinion was long entertained vaguely by many physicians that small-pox and vaccinia were entirely distinct, though similar, diseases. This belief was based upon the fact that Jenner did not himself fully comprehend the manner in which these two forms are related. He believed that they were identical in their origin, but he did not demonstrate that identity."

The opinion is still held by eminent vaccinologists of the non-identity of variola and vaccinia. Recent investigations upon the subject have tended to confirm the opinion that the diseases are structurally and clinically different. Surely, in the estimation of most excellent students, the beautiful experiments of the late lamented Ceely, as to the production of vaccinia by the inoculation of cows with variolous matter, are seriously doubted. These experiments by Ceely, of all the series now so famous in the history of vaccination, were the most reliable and convincing, although we think we can show that good reasons exist why we should regard them with distrust, if not reject them entirely. We contend that small-pox and cow-pox are not identical:

1. Because cow-pox has never been converted by any means into small-pox.

2. Because, in the light of the attempts made by many careful and truthful experimenters, to produce small-pox by the inoculation of the cow with small-pox virus, the number of failures on the part of such a very large majority of them, together with the numerous positive denials made of the possibility of success, it may be concluded that small-pox virus is never converted into vaccine by transmission through the cow. The results of Gassner, of Ceely, and Thielé, and Badcock are not left out of consideration in making this statement.

3. Cow-pox does not become epidemic,* nor does it spread by effluvia from the human subject. It is always confined to the

* An Inquiry into the Causes and Effects of Variolæ Vaccinæ. Edward Jenner, M. D., F. R. S., Springfield Edition, 1802, pp. 54 and 55. "It (cow-pox) has been conceived to be contagious among cows without contact; but this idea cannot be well founded, because the cattle in one meadow do not infect those in another, unless they be handled or milked by those who bring the infectious matter with them."

point of insertion in the human subject, never producing a general eruption of vaccine vesicles.

4. Structurally considered, the vesicles of vaccination and the pustules of inoculation are very dissimilar. Clinically considered, the course and duration of the two diseases are not unlike, but certainly not identical, having enough points of difference to enable the skilled diagnostician to differentiate them.

It becomes necessary, in order that we shall not do injustice to the experimenters who have claimed to produce genuine cow-pox by the inoculation of cows with small-pox virus, to go over the history of these attempts as briefly as possible.

The impulse given to these attempts was by Jenner's theory, that vaccine was but a species of small-pox. His nomenclature fully proves this: thus, he names the vaccine disease *variolæ vaccinæ.* Moreover, he fully enunciates his belief in the identity of small-pox and cow-pox, and his biographer says: * "He always considered small-pox and cow-pox as modifications of the same distemper; and that in employing vaccine lymph, we only made use of means to impregnate the constitution with the disease in its mildest, instead of propagating it in its virulent and contagious form." † There has been always a traditional opinion of the identity of cow-pox, founded upon the statements of writers, contemporaries of Jenner, or following closely after him. There was little originality in any of the volumes advocating this theory, many of them following Jenner's narrative precisely, adding only some cases coming under their observation.

None of the variolous inoculations of cattle for artificial cow-pox claimed to be successful ‡ until 1807, nine years after Jenner gave his "Inquiry" to the world,—the first attempt reported as being successful, being performed by Dr. Gassner, of Günzberg. The results were called in question, says Trousseau.§ Twenty-nine years later, Thielé, of Kasan, in Russia, announced successful production of artificial cow-pox. The small-pox matter em-

* Baron's Life of Jenner, p. 241.

† The theory of the transmission of grease from the horse to the cow was the favorite theory of Jenner. Inquiry, pp. 58 and 59.

‡ These inoculations had been tried in vain by Coleman, Ring, Sacco, Numann, Fiard, Bousquet, Dalton, etc. *Steinbrenner*, Traite Sur la Vaccine, p. 613.

§ Lectures on Clinical Medicine. Vol. II. English Edition, p. 115.

ployed in these inoculations was taken immediately from variolous pustules, while they were yet transparent, nacreous, pearly, and of which the lymph was still very limpid; or, better still, he used lymph which had been kept ten to twelve days between two pieces of glass.* Steinbrenner, in remarking on the results of Thielé, says: "Let us say furthermore, *en passant*, that this author, convinced by his experience of the analogy of the vaccine and variolous virus, had the idea of trying to mitigate the latter, in order to make it produce the same results as vaccine, and that without making it pass previously through the body of the cow. He pretends it will become so by leaving variolous virus for ten or twelve days between two glasses closed with wax; then he only dilutes it with tepid milk, and uses it thus prepared. He repeated the same thing with new lymph for many [series] following. The secondary fever did not show itself; the virus becomes so mitigated in the sixth generation that it produces nothing more than the symptoms of ordinary vaccine. It can then be employed without further precautions to vaccinate from arm to arm." (Steinbrenner, p. 615.)

It is very evident from the above statement that if Thielé's observations as to variolous inoculation are no sounder than his opinion of extracting the virulency from small-pox virus, that we must be exceedingly guarded how we accept his opinion. In the light of our present knowledge, it would be the height of folly to dare to go before the public with such an announcement. Small-pox virus cannot be tamed by such gentle means; you may destroy it utterly, but so long as there is any activity left it maintains its same contagious properties, and probably cannot be so diluted as to be harmless. It is true, there are degrees of small-pox. Some types are so mild as to appear harmless. I inoculated one patient with small-pox virus in 1865, and he had only two resulting pustules, although there was no evidence of vaccination, judging by absence of cicatrices. But can any one deny that an unprotected person coming in contact with the mildest cases of small-pox; one like this, for instance, would not be liable to small-pox in some form?

---

* Steinbrenner, p. 614.

Steinbrenner himself also failed to inoculate two cattle upon which he made the attempt, although he says that he was not then informed of the minutiæ of the process as detailed by Thielé and Ceely.

Dr. Ceely's cases are not so easily disposed of by merely denying his success. Dr. George Gregory * was willing to admit Ceely's success. "No doubt," he says, "can be entertained of their correctness." * * * "But "the lymph thus obtained has been called variolo-vaccine, to distinguish it from the idiopathic affection of the animal." "One effect of these experiments (of Ceely's) has been to refute Jenner's favorite notion, that the cow-pox is the parent of small-pox. So far as they go, they tend to show that small-pox is the primary, and cow-pox the secondary disorder. But it may be reasonably asked, do these experiments warrant the conclusion that cow-pox and small-pox are identical? To me it appears that they do not. The disorders are allied (so are measles and scarlatina), but they are not therefore identical. * * * Unlike small-pox, cow-pox produces no eruption, no constitutional disturbance; it throws off no contagious emanations. It can be perpetuated from man to man in a uniform state of intensity; whereas the inoculation of small-pox produces the disorder in varying shades of severity. The local characters of each malady are no less strikingly contrasted. The variolous action goes on to pustulation, to the *acumination* of the pustule, to sloughing of the corion, and implication of the subjacent cellular membrane. The vesicle of cow-pox never loses its umbilicated character; no purulent matter forms; the areola is circular, not irregular, like that of the inoculated small-pox."

The above quotations are made from this well-known master, who for years was physician to the Highgate Small-pox Hospital, and who was a teacher of recognized ability at St. Thomas' Hospital. These opinions were expressed only a few years after Ceely's famous experiments were published.

When we consider these inoculations in the light of our present knowledge of the natural history of cow-pox as observed in the large number of vaccinations directly from the cow, it is plainly

* Gregory, Lectures on "*The Eruptive Fevers.*" Am. Edition, 1851, p. 265.

evident that the matter obtained by Ceely was quite different from the animal virus of the Beaugency stock, now so well known in the United States.

It will be observed that writers upon the use of vaccine directly from the cow, especially as to the use of variolo-vaccine, give warning that a more violent form of vesicle would result from the insertion of such lymph ; or, they instruct the vaccinator that frequent failures might be expected.

Dr. Seaton says (Handbook of Vaccination, p. 87): "Ceely found that more than half his attempts to vaccinate with primary cow-lymph, taken from vesicles at a proper stage, and possessing all the characteristics of perfection, resulted in entire failure, the same individuals being successfully vaccinated immediately afterward with the current humanized vaccine lymph."

With our knowledge of the course of a vaccination from the current Beaugency stock of animal virus, it is not difficult to believe that this virus which behaved so uncertainly had little relationship to the certain, normal, active stock now propagated in this country. All along through the history of the Ceely virus we read of uncertainties, of abnormal actions, and of suspicious phenomena, which virtually rendered its use impracticable, and which gave little encouragement for others to follow, except as a *dernier ressort.*

A new chapter on the production of artificial cow-pox was worked out during the late war, in the Confederate States. The question of the supply of genuine vaccine became such a serious one, that medical officers were commissioned to undertake the study of the immense number of spurious vaccinations, and devise the remedy. I speak within bounds when I say that in this country it was rare to find a medical man who was at all skeptical as to the possibility of procuring artificial vaccination at will. The experiments of Ceely were studied with peculiar interest, as were those of Sonderland. After attempting again and again to restore the efficacy of vaccine by careful cultivation among country children, no adequate supply was obtained, and then the variolation of cattle, by the Ceely and Sonderland method, was resorted to. Dr. James Bolton, late of Richmond, Va., undertook the experiments for the production of artificial cow-pox. He says:* "I made experiments upon seven animals during the war. Five of them were young cows, or heifers, which were furnished me by the Commissary Department, by order of the Surgeon-General. They were kept at Howard's Grove, in the immediate vicinity of the Small-Pox Hospital. From the latter place I procured variolous virus in the form of lymph and crust. This I inserted into the udder by making light incisions, and rubbing into them while in the liquid state, by dipping threads into the lymph and passing them by means of a needle under the integument; and finally by making punctures with a lancet and introducing pieces of crusts; but all these attempts failed. In two instances pustules were produced, followed by thin yellow crusts, which were evidently spurious results." * * * "I have my doubts that genuine vaccine is obtained by inoculating cows with small-pox virus." Subsequently Dr. Bolton wrote me: "I have my doubts that the small-pox virus can be inserted at any part of the udder from which it cannot be licked off. The tongue of the cow can be protruded so far, and can be drawn into such anomalous curves, with such prehensile power, that I think any part of the udder can be reached." The details of Dr. Bolton's experiments were destroyed by the casualties of war, and his account was written from memory.

Dr. S. P. Crawford, of Greenville, Tenn., writes to Prof. Joseph Jones, M.D.:† "About this time" (January, 1865) "orders were issued from Surgeon-General Moore to procure fresh vaccine virus through the cow. I tried the experiment three times, without succeeding. First on a cow seven years old, with a calf six months old; then a young heifer two years old, and finally the calf. I inserted the matter in the teats, nose, ears, and various parts of the skin, but never succeeded in getting a crust. Finally I fed the calf on the dried scabs, but it died without yielding the much sought treasure."

Dr. William J. Love, of Wilmington,‡ "had occasion to at-

* Non-Identity of Vaccinia and Variola, by Thomas F. Wood, M.D., 1867, p. 17.

† Researches Upon "Spurious Vaccination" During the American Civil War. By Joseph Jones, M.D. Nashville, 1867, p. 99.

‡ Non-Identity of Vaccinia and Variola, p. 17.

tempt the same experiment" [variolous inoculation of a cow], "and failed." He introduced the small-pox virus into the teats and udder, by means of a saturated thread. He also made incisions a little deeper than in the human subject for like purpose, and introduced the lymph in this way. Finally he smeared collard leaves with the lymph, and fed the cow on them. He saw no other phenomena, except such as would result from the insertion of any foreign matter,

I had occasion just after the war (1865–'66), while in charge of the Wilmington Small-Pox Hospital during an epidemic of the disease, to go over the same ground of attempting the production of artificial cow-pox. My experiments were done without any knowledge on my part of the details of the operation, except such as I had received through the gentlemen above quoted, and by the perusals of Ceely, Thielé's and Sonderland's experiments. It is not necessary to detail these experiments,, as they differed in no respect, as far as I was able to judge, from the description given by these authors. The failure was complete, in my opinion.

It happened, though, that during the progress of the experiment, that an army medical inspector, whose name I have forgotten, was making a tour of the hospitals, hearing of my experiments, visited my hospital, and after examination pronounced the small vesicles genuine cow-pox, and confirmed his faith in his opinion by making some inoculations on the arms of two children in an Irish family near by. The inoculation resulted in a genuine small-pox, which went through the family in various grades of intensity.

A reviewer in the *Medical Times and Gazette*, July 2, 1881, discussing Mr. Fleming's recent work, "Human and Animal Variolæ," says: "It seems to be commonly held among medical men in this country that cow-pox is not a disease natural to the bovine species, but merely small-pox conveyed to them from man by inoculation, and that in the operation of vaccination we avail ourselves of the modification which it has undergone in the body of the cow, precisely as Dr. Greenfield confers immunity from anthrax on cattle by inoculation with bacilli which have passed through the system of the rabbit.

"In support of this view, it is usual to adduce the supposed rarity of cow-pox now that small-pox has become almost unknown in rural districts, the asserted exemption of the male sex among cattle, the successful inoculation of cattle with small-pox, with the production of what Thielé and Ceely believe to be cow-pox, and the vaccination of infants therefrom, by the same observers.

"Mr. Fleming has collected a vast amount of evidence from French, German, Scandinavian and Italian sources, and has carefully scrutinized the scanty English experimenters. He gives irresistible evidence of the prevalence of cow-pox in countries where small-pox is almost unknown,* of the equal susceptibility of bulls, if exposed to the contagion with cows, and satisfactorily proves that the supposed vaccinations of Mr. Ceely were, in fact, nothing more or less than an inoculation with small-pox unchanged by its transmission through the cow. Such inoculation of the cow is not often successful, but in several instances the real nature of the lymph thence taken and implanted in children has been manifested not only by the eruption following, but by the communication of unmistakable and fatal small-pox to other infants by ordinary infection—a caution to those who would attempt to reproduce a more active vaccine through such a procedure as mediate variolation. * * * The disastrous results of mediate variolation instead of vaccination, in America and India, show the value of a knowledge of the diseases of domestic animals."

The *opinions* as to the non-identity of vaccinia and variola are very numerous, and writers are about equally divided, perhaps. It possibly may not be definitively settled in our day, and indeed, the question of identity may be no longer a practical one. This will depend greatly upon the way in which the medical profession makes use of the triumphant solution of the question as established in the introduction of animal virus by Dr. Henry A. Martin. It does not seem possible that with our present enlightenment, a vaccine famine can occur in this country, and for years no more attempts will be made to produce artificial cow-pox. Never-

* Cow-pox has appeared in communities where there has been no small-pox at the time. A recent most convincing series of cases were reported by Dr. Henry A. Martin, of Boston, at the last meeting of the American Medical Association. These cases of spontaneous cow-pox occurred in 1880, in Massachusetts, and their relation was rather to horse-pox than small-pox, a case of the former having also occurred in the same neighborhood.

theless, the warning should go forth authoritatively, that the production of artificial vaccination is a myth, and in the times of great danger from variolous epidemic, the time when it is most eagerly desired to do something effectual, it has most signally failed.

Let the decision be what it may as to the theory of identity, it is practically valueless, and always dangerous, both on account of the delay, and of the risk of pure variolous inoculation. Those who have claimed success have left such a record of difficulties to overcome, and disappointments to suffer, that we can no longer have faith in it as a source of vaccine, even though we should not discredit the truth of identity.

I had intended to discuss more fully the points in Dr. Lyman's paper on vaccination, above referred to, but I have overstepped the bounds in consideration, mainly, of the question of identity. I am pleased to see that we agree as to efficacy of animal virus. I have nothing but praise for the results of vaccination done with Beaugency virus. I trust that the potency of this virus may forever prevent the adoption of Dr. Lyman's suggestion: "The only efficient remedy for this deterioriation [referring to the possibility of the degeneracy of bovine virus] consists in occasional reversions to the original product of the variolated cow." I am convinced that more diligent searching by experts will detect now and again genuine cow-pox. From this source alone will the pure succession be maintained. Knowledge is accumulating very greatly upon the natural history of cow-pox, and a widespread interest will surely manifest itself among intelligent farmers, making the mishap of the non-occurrence of cow-pox less and less probable.

